# An Acyl-CoA Synthetase in *Mycobacterium tuberculosis* Involved in Triacylglycerol Accumulation during Dormancy

**DOI:** 10.1371/journal.pone.0114877

**Published:** 2014-12-09

**Authors:** Jaiyanth Daniel, Tatiana Sirakova, Pappachan Kolattukudy

**Affiliations:** 1 Burnett School of Biomedical Sciences, University of Central Florida, Orlando, Florida, 32816, United States of America; 2 Department of Biology, Indiana University-Purdue University Fort Wayne, Fort Wayne, Indiana, 46805, United States of America; Centre National de la Recherche Scientifique - Université de Toulouse, France

## Abstract

Latent infection with dormant *Mycobacterium tuberculosis* is one of the major reasons behind the emergence of drug-resistant strains of the pathogen worldwide. In its dormant state, the pathogen accumulates lipid droplets containing triacylglycerol synthesized from fatty acids derived from host lipids. In this study, we show that Rv1206 (FACL6), which is annotated as an acyl-CoA synthetase and resembles eukaryotic fatty acid transport proteins, is able to stimulate fatty acid uptake in *E. coli* cells. We show that purified FACL6 displays acyl-coenzyme A synthetase activity with a preference towards oleic acid, which is one of the predominant fatty acids in host lipids. Our results indicate that the expression of FACL6 protein in *Mycobacterium tuberculosis* is significantly increased during *in vitro* dormancy. The *facl6*-deficient *Mycobacterium tuberculosis* mutant displayed a diminished ability to synthesize acyl-coenzyme A in cell-free extracts. Furthermore, during *in vitro* dormancy, the mutant synthesized lower levels of intracellular triacylglycerol from exogenous fatty acids. Complementation partially restored the lost function. Our results suggest that FACL6 modulates triacylglycerol accumulation as the pathogen enters dormancy by activating fatty acids.

## Introduction

Latent infection with *Mycobacterium tuberculosis* (*Mtb*) affects two billion people worldwide [1], [2]. In latent TB, *Mtb* is in a dormant state and becomes phenotypically tolerant to antibiotics, loses acid fastness and accumulates triacylglycerol (TAG)-containing lipid droplets for possible use as energy source during dormancy and reactivation [3]–[6]. The phenotypic drug tolerance of dormant *Mtb* prolongs the treatment regimen required to cure TB, results in poor compliance and contributes to the emergence of multi-drug resistance [2].

Host-derived fatty acids are critically important as energy source during *Mtb* dormancy [4], [7], [8] but the metabolic pathways utilized by *Mtb* for activating host fatty acids destined for bacterial TAG accumulation as it enters dormancy remain unexplored. Fatty acid transport proteins (FATPs) appear to be critical players in the transport of fatty acids across cell membranes [9]–[11]. FATPs are integral membrane proteins with two conserved domains: an ATP/AMP binding domain that is conserved from bacteria to man and a fatty acid binding domain unique to FATPs [12], [13]. The long-chain acyl coenzyme A synthetase (ACSL) activity of the FATP, which mediates the activation of fatty acids, probably plays an important role in regulating the rate of fatty acid uptake and in channeling the imported fatty acids between various metabolic processes within the cell [13], [14]. Thus ACSLs are thought to act as “metabolic sinks” that drive fatty acid transport across membranes [15]. Furthermore, ACSLs are thought to be involved in the sequestration of fatty acids into distinct pools within the cell for diverse metabolic purposes [13], [16]. Mammalian FATP1 has been demonstrated to channel exogenous fatty acids into triacylglycerol biosynthesis [17]. Thus, it is possible that a mycobacterial ACSL may play a critical role in the sequestration of fatty acids for TAG synthesis within *Mtb*.

We postulate that mycobacterial ACSL proteins may be involved in utilizing host lipid-derived fatty acids for the synthesis of TAG inside *Mtb* during dormancy. The *Mtb* genome encodes 34 fatty acyl-CoA ligase [FACL]-like gene products and some of them synthesize acyl-AMP for use by polyketide synthases [18]. However, Rv1206 (FadD6/FACL6) is the only member of the family that belongs to a family of fatty acid transporters conserved across several species [12]. In this study, we show that the purified FACL6 protein displays acyl-coenzyme A synthetase activity and is able to indirectly stimulate fatty acid uptake in *E. coli* cells. We found that the FACL6 protein level was higher in *Mtb* cells in a dormant state than in *Mtb* cells in exponential growth phase. We constructed an *Mtb* mutant lacking FACL6 and found that the deletion of FACL6 resulted in a significant decrease in the accumulation of intracellular TAG in *Mtb* under dormancy-inducing conditions *in vitro*. Complementation partially recovered the lost phenotype.

## Materials and Methods

### Chemicals and reagents

[^14^C]Oleic acid (56 Ci mol^−1^) was purchased from American Radiolabeled Chemicals, Inc. All other chemicals and media were purchased from Sigma (St. Louis, MO) and Thermo Fisher Scientific Inc. Nucleotide primers were synthesized by Integrated DNA Technologies, (Coralville, IA).

### Bacterial strains and growth conditions

*M. tuberculosis* (*Mtb*) H37Rv, *facl6* (*Rv1206*) deletion mutant of *Mtb* (d-FACL6) and *facl6*-complemented strain of d-FACL6 (C-FACL6) were grown in Middlebrook 7H9 broth (supplemented with 0.05% Tween 80, 10% oleic acid-albumin-dextrose-catalase enrichment, and 0.2% glycerol) up to OD_600_ nm of 0.6. The strains were stored at −80°C as stock cultures with 15% (v/v) glycerol. The d-FACL6 and C-FACL6 strains were grown in media containing hygromycin (Hyg, 75 µg ml^−1^) and Hyg (75 µg ml^−1^) plus kanamycin (Kan, 30 µg ml^−1^) antibiotics respectively. The *Mtb* strains were subjected to multiple-stress conditions as we have described previously [19]. Briefly, *Mtb* cultures in log-phase were exposed to a combination of stresses that are thought to be experienced by the pathogen in the human body (low [5%] oxygen, high [10%] carbon dioxide, low pH [5.0] and nutrient starvation [10% Dubos medium]). Luria-Bertani broth was used for all *E. coli* cultures, and when required, antibiotics were added to the culture at the following concentrations: kanamycin, 50 µg ml^−1^; carbenicillin, 50 µg ml^−1^.

### Multiple-sequence alignment

The amino acid sequence of *M. tuberculosis* FACL6 (Rv1206; Gene ID: 13319785) was aligned with human FATP1 (HsFATP1; Gene ID: 376497), human FATP4 (HsFATP4; Gene ID: 10999) and yeast FATP (ScFAT; Gene ID: 852329) amino acid sequences using the ClustalW2 multiple sequence alignment program (http://www.ebi.ac.uk/Tools/msa/clustalw2/). The alignment was shaded using the BoxShade program (http://www.ch.embnet.org/software/BOX_form.html) and manually adjusted for optimal alignment.

### Cloning and expression of recombinant FACL6

**Figure 1:**
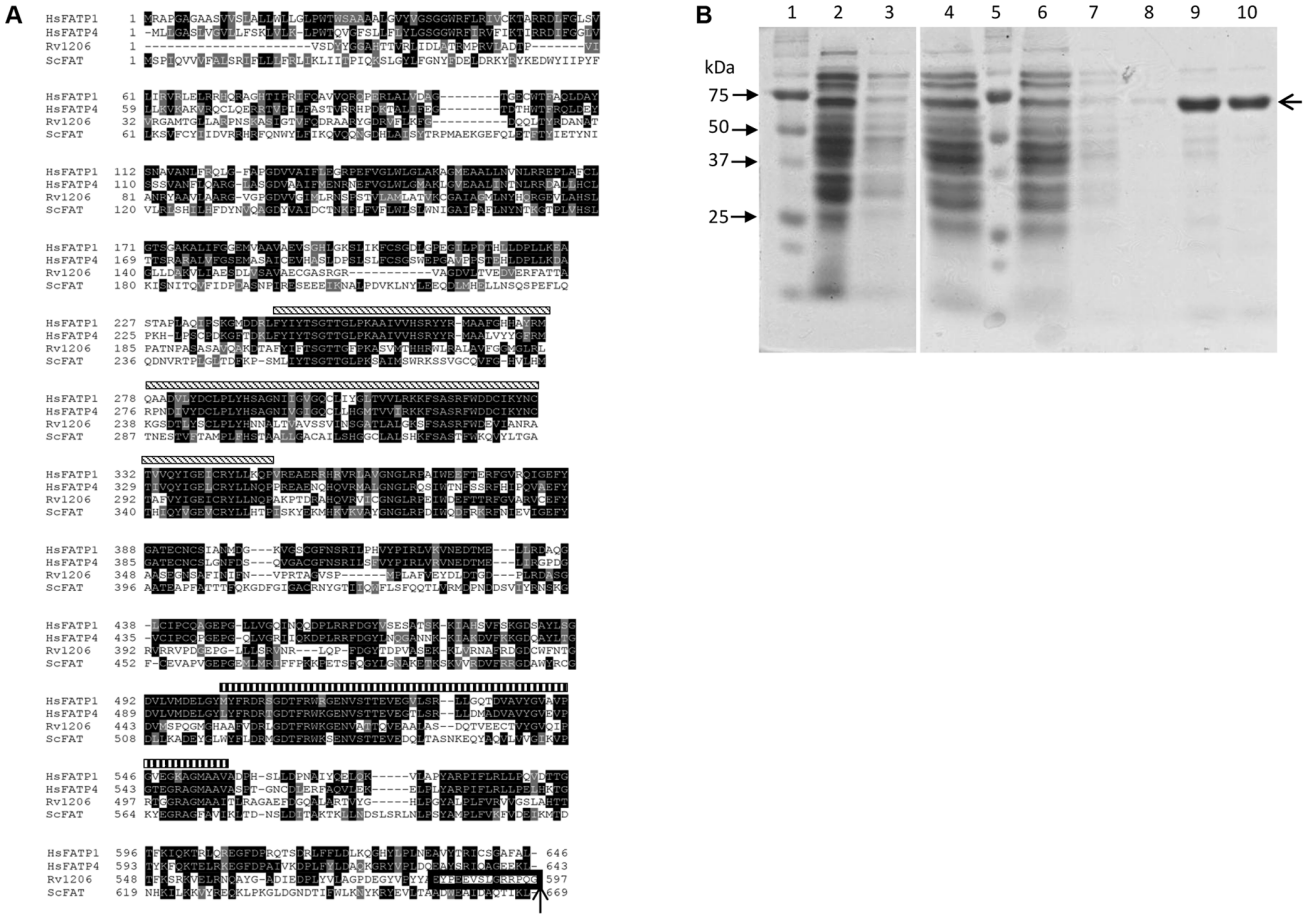
Alignment of FACL6 amino acid sequence with eukaryotic FATPs and purification of FACL6. **A,** The FACL6 (Rv1206) amino acid sequence was aligned with human FATP1 (HsFATP1), human FATP4 (HsFATP4) and yeast FATP (ScFAT) amino acid sequences using the ClustalW2 multiple sequence alignment program. Amino acid identities are shaded in black and similarities shaded in gray. The diagonally hatched boxes above the amino acid sequences indicate the location of the AMP-binding domain and the vertically hatched boxes indicate the location of the very long-chain acyl-CoA synthetase (VLACS) domain on the human FATP1. The peptide indicated by a box and arrow at the C-terminal end of Rv1206 (FACL6) was used to raise a polyclonal antiserum against FACL6. **B,** Purification of FACL6 expressed in *E. coli*. The FACL6 protein was solubilized and affinity-purified as described in Materials and Methods. Coomassie-stained denaturing polyacrylamide gel shown. Lanes: 1 and 5, molecular weight standards (sizes indicated on left); 2, cell lysate; 3, 16,000×g supernatant; 4, supernatant from solubilized 16,000 x g pellet; 6, column flow-through; 7 and 8, column wash; 9 and 10, eluted fractions containing purified FACL6 protein.

The *facl6 (Rv1206)* ORF corresponding to the protein shown in Fig. 1 was PCR-amplified from the genomic DNA of *Mtb* H37Rv using *Pfu* Turbo HotStart DNA polymerase (Agilent Genomics, CA) and cloned into pET200 D-TOPO expression vector (Life Technologies, NY) for expression of N-terminal histidine-tagged FACL6 in *Escherichia coli* BL21 Star (DE3) cells. Sequence integrity was confirmed by DNA sequencing. Protein expression was induced for 4 h at 37°C by adding IPTG (isopropyl-β-D-thiogalactopyranoside) to a final concentration of 1 mM when the culture reached an optical density at 600 nm (OD_600_) of 0.6. The FACL6 protein was also expressed as a C-terminal histidine-tagged fusion protein in the pBAD/*Myc*-His vector (Life Technologies, NY) under the control of the *araBAD* promoter in *E. coli fadLfadR* mutant LS6164 [20], [21]. All subcloning procedures were carried out in pCR Blunt II TOPO vector (Life Technologies, NY) in *E. coli* TOP10 cells (Life Technologies, NY). Expression of FACL6 in *E. coli* mutant LS6164 at OD_600_ of 0.6 was induced for 4 h by the addition of arabinose to a final concentration of 0.2% (w/v).

### Purification of FACL6

Expression of FACL6 in pET200 D-TOPO construct was induced with IPTG in a 600 ml culture of *E. coli* BL21 Star (DE3). The cells were washed and resuspended in lysis buffer (50 mM sodium phosphate pH 7.4, 300 mM NaCl with a cocktail of protease inhibitors) and were disrupted by sonication using a Branson Sonifier 450 (Branson Ultrasonics Corp.). The cell lysates were clarified by centrifugation at 16,000 x g, 4°C and the pellet was used for purification of the expressed protein since our attempts to purify the protein from supernatant were unsuccessful. The pellet was solubilized in 5 ml ice-cold lysis buffer with 1% (w/v) dodecylmaltoside and 0.03% Triton X-100 for 15 min with intermittent sonication to enhance solubilization of the inclusion bodies. The solution was centrifuged and the supernatant was used to purify the FACL6 protein using a 5-ml bed volume TALON cobalt-affinity resin (Clontech, CA) according to the manufacturer’s protocol. The column was washed with 10 mM imidazole and bound protein was eluted with 200 mM imidazole. The proteins in each fraction were resolved by sodium dodecyl sulfate-polyacrylamide gel electrophoresis and visualized by coomassie staining.

### Acyl coenzyme-A synthetase assay

The purified FACL6 was assayed for acyl coenzyme-A (acyl-CoA) synthetase activity following a modified protocol of Nambi et al. [22]. Briefly, the assay mixture, in a 40 µl reaction volume, contained about 7.5 µg purified FACL6 protein, 100 µM ^14^C-oleic acid (or ^14^C-palmitic acid or ^14^C-stearic acid or ^14^C-hexacosanoic acid), 5 mM ATP, 2 mM CoA, 2.5 mM MgCl_2_, 0.005% (v/v) Triton X-100 in 50 mM Tris-HCl pH 8.0. After incubation for 15 min at 30°C, the reaction was stopped by addition of 2.5 µl glacial acetic acid. The entire reaction mix was loaded onto a silica-thin layer chromatography (TLC; Analtech, Newark, DE) plate that was resolved using *n*-butanol-acetic acid-water (15∶5∶8, by volume) as the solvent system. Acyl-CoA product migrated with an R_f_ of 0.55 while fatty acid substrate migrated with an R_f_ of 1.0 (near the solvent front). The TLC plate was imaged by autoradiography and radioactivity in acyl-CoA band was measured by liquid scintillation counting to obtain a quantitative measure of acyl-CoA synthetase activity.

Acyl-CoA synthetase activity in cell-free extracts (prepared by bead-beating *Mtb* cells from 10 ml cultures in 0.5 ml ice-cold 100 mM Tris-HCl pH 7.5 containing protease inhibitor cocktail) of *Mtb* subjected to dormancy-inducing multiple stress was assayed using a protocol modified from Hall and colleagues [23]. Briefly, cell-free extracts of *Mtb* containing about 50 µg of protein were incubated in a reaction mix containing 100 mM Tris-HCl pH 7.5, 50 µM ^14^C-oleic acid (4 Ci mol^−1^), 10 mM ATP, 5 mM MgCl_2_, 200 µM CoA, and 200 µM dithiothreitol and 0.1% (v/v) Triton X-100. After incubation at 37°C for 30 min, reactions were stopped by adding 1.25 ml isopropanol:hexane:sulfuric acid (40∶10∶1, by volume) followed by addition of 0.5 ml water and 0.75 ml hexane. After vortexing and brief centrifugation to separate the organic phase, the aqueous phase was removed and further extracted thrice with 0.75 ml hexane to remove unreacted ^14^C-oleic acid. Radioactivity in aqueous phase representing acyl-CoA synthesis was determined by liquid scintillation counting.

### Radiolabeled fatty acid uptake assay

A 10 ml culture of *E. coli* LS6164 transformed with the pBAD-FACL6 expression plasmid was induced with arabinose, as described above. Untransformed cells were used as negative control. Expression of the FACL6 protein is expected to increase cell-associated radioactivity. Since the LS6164 mutant was shown to be defective in fatty acid uptake [20], [21], the background radioactivity levels are expected to be low in the untransformed controls. Therefore, we selected this mutant for expressing the FACL6 protein. Following induction with arabinose for 4 h, OD_600_ of untransformed and transformed cells was measured. Using OD_600_, cell numbers of untransformed and transformed cells were equalized by pelleting appropriate volumes by centrifugation. The cells were then washed with pre-warmed fatty acid uptake buffer (phosphate-buffered saline [PBS] containing 0.1% [w/v] fatty acid-free bovine serum albumin). The cells (from approximately 2 ml of induced culture) were then resuspended in 1 ml of fatty acid uptake buffer containing 37 µM (2 µCi) ^14^C-oleic acid and incubated with gentle agitation at 37°C. At the indicated time-points, 0.05 ml of cell suspension (in duplicate) was centrifuged for 1 min at 16,000×g and the cell pellet was washed thrice with ice-cold fatty acid uptake buffer without radiolabel to remove extracellular radioactive fatty acid and stop the uptake. The cell pellets were then resuspended in PBS and the radioactivity associated with cells was determined by liquid scintillation counting.

### Western blotting analysis of FACL6 protein levels

A C-terminal epitope of FACL6 (EYPEEVSLGRRPQG; indicated in Fig. 1) was used to generate a polyclonal antiserum in rabbits (Thermo Scientific Pierce Custom Antibody Services, Rockford, IL). The anti-FACL6 IgG was purified from antiserum using Melon Gel IgG Purification Kit (Thermo Scientific, Rockford, IL). Cell-free lysates were prepared as described above from *Mtb* wild type cultures in log-phase or after 18 days under multiple-stress from two independent experiments. The lysates were equalized by total protein content (20 µg protein each) since dormancy-associated down-regulation in overall protein synthesis in *Mtb* cells made the use of a single loading control protein for log-phase and dormancy-induced *Mtb* cells impractical. Lysates were resolved by 12% denaturing polyacrylamide gel electrophoresis and analyzed by Western blotting. The primary antibody (anti-FACL6 IgG) was used at a dilution of 1∶500 and the secondary antibody (mouse anti-rabbit IgG HRP; Santa Cruz Biotechnologies, Dallas, TX) was used at a dilution of 1∶5000. Blot hybridization buffer was phosphate-buffered saline (PBS) containing 5% bovine serum albumin and wash buffer was PBS containing 0.05% (v/v) Tween-20. Blots were incubated with SuperSignal West Dura Substrate (Thermo Scientific, Rockford, IL) and imaged by exposure to photographic film.

### Generation of facl6 gene-deletion mutant of Mtb

Genomic DNA of *Mtb* H37Rv was isolated by the guanidine thiocyanate method as reported [24]. The disrupted mutants were constructed by allelic exchange via specialized transduction using the temperature sensitive mycobacteriophage phAE159 as previously described [25]. The allelic exchange by double crossover was confirmed with two sets of primers (H1 and H2), each representing a hygromycin (hyg) primer, and primers in the mycobacterial genome outside the gene sequence used for making the disruption construct, G1 and G2 (Table 1). The deletion mutants were selected and confirmed as previously described [25]. For Southern blot hybridization, DNA samples were digested with PstI, subjected to 1% agarose gel electrophoresis, transferred to nylon membranes (Nytran Plus; Schleicher and Schuell), and hybridized with [α-^32^P] dCTP-labelled probes using the random prime labeling system Rediprime II (Amersham Pharmacia).

**Table 1:** PCR primers used for *facl6* disruption in *Mtb.*

**Primer pairs for amplification of 5′ and 3′-flanks of *facl6***
**5′-flank**
*facl6*-A: 5′-CTTAAGACATAGACCTAGCTCGGCAGCC-3′
*facl6-*B: 5′-TCTAGAGTAGTCCGCCAACACTCGCGG-3′
**3′-flank**
*facl6*C: 5′- AAGCTTCACACCACGACGTTCAAGAGTCGC-3′
*facl6*D: 5′-ACTAGTTCACAGCATCCACCACACCGCG-3′
**Primer pair for amplification of sequence inside the deleted segment**
*d-facl6*-F: 5′-GCGATTCGCCACAACGGCGCC-3′
*d-facl6*-R: 5′-GGGTCGGTAGTAGCCGTCGAACGGC-3′
**Primer pairs for amplification of genomic flanks in the mutants**
**5′-flank**
G1∶5′-AAAGCGCCGAACGGGATAGGCCC-3′
H1∶5′-TAGAGGCGATAGGTAGGTAGTCGATAGCT-3′
**3′-flank**
H2∶5′-GGAACTAGGCGCAGTTCCTCTAGGGG-3′
G2∶5′-GCCGGGTCGATCAACACCTCTCGCG-3′
**Primer pair for amplification of *facl6* ORF for complementation using pMV361**
Forward: 5′-AAGCTTTAGTGTCCGATTACTACG-3′
Reverse: 5′-AAGCTTCTAGCCCTGCGGTCGCC-3′

### Complementation of the facl6-deletion mutant of Mtb

The *facl6* coding sequence amplified from *Mtb* H37Rv genomic DNA and cloned into the HindIII site of the integrative mycobacterial expression vector pMV361 was used to complement the *facl6*-deletion mutant as we have described earlier [24]. The primers used to amplify the open reading frame the sequences are indicated in Table 1.

### Incorporation of fatty acids into lipids by d-FACL6 mutant of Mtb

*Mtb* wild type (WT), FACL6-deletion mutant (d-FACL6) and complemented FACL6 mutant (C-FACL6) were subjected to dormancy-inducing multiple-stress conditions in media without Tween-80 following procedures we have previously described [19]. The cells were then labeled with ^14^C-oleate (55 Ci mol^−1^; 5 µCi per 10 ml culture; Perkin Elmer, Waltham, MA) for 4 h and incorporation of radiolabel into TAG and polar lipids was determined. The accumulation of TAG by the cells was investigated by incubating the multiple-stressed *Mtb* cultures with oleic acid (100 µM; Sigma-Aldrich, St. Louis, MO) for 1 additional day under the same conditions. The *Mtb* lipids were extracted using chloroform/methanol (2∶1, by vol.) and equal volumes of the lipid extracts were resolved on silica-TLC using hexane-ethyl ether-formic acid (40∶10∶1, by vol.) as solvent system. Authentic lipid standards were resolved on the same TLC plates as the experimental samples and visualized under UV light after spraying them with 0.1% (w/v) solution of 2′,7′-dichlorofluorescein in 95% (v/v) ethanol. The TLC plates were subjected to autoradiography to image the radiolabeled lipids. Bands corresponding to TAG and polar lipids (PL; origin on TLC plate) were scraped from the TLC plate and radioactivity was determined by liquid scintillation counting. In order to control for sample-to-sample variations in radiolabel incorporation, a normalization of radioactivity in lipid classes in wild-type and *d-facl6* mutant was done as follows: Radioactivity in TAG or PL was determined and normalized across samples by using the radioactivity in total lipid extract in the respective sample as the denominator for each sample. The results of such normalization are shown as a percent of radioactivity in respective total lipid extract. Non-radiolabeled lipids were charred at 180°C after spraying the TLC plate with dichromate-sulfuric acid and band intensity was quantitated by densitometry using a gel-documentation system.

## Results

### Sequence similarity of FACL6 with eukaryotic fatty acid transport proteins

An open reading frame in *Mtb* has been suggested to encode a protein belonging to a family of FATPs conserved from mycobacteria to humans [12]. Although this gene (Rv1206) is annotated as a probable fatty acyl-CoA ligase (fadD6), it was shown to stimulate fatty acid uptake in *E. coli* [12]. As suggested by others earlier [18], we refer to this protein as FACL6. We aligned the amino acid sequence of FACL6 with the amino acid sequences of human FATP1, human FATP4 and yeast FATP using the ClustalW2 multiple sequence alignment software and the alignment is shown in Fig. 1. The human and yeast FATPs have been studied extensively, their AMP-binding and very long-chain acyl-CoA synthetase domains have been previously identified [13], [14]. Multiple sequence alignment indicates that FACL6 contains domains homologous to these domains and we have indicated their locations on the multiple sequence alignment by hatched bars (Fig. 1A).

### Purified FACL6 protein displays acyl-CoA synthetase activity

**Figure 2:**
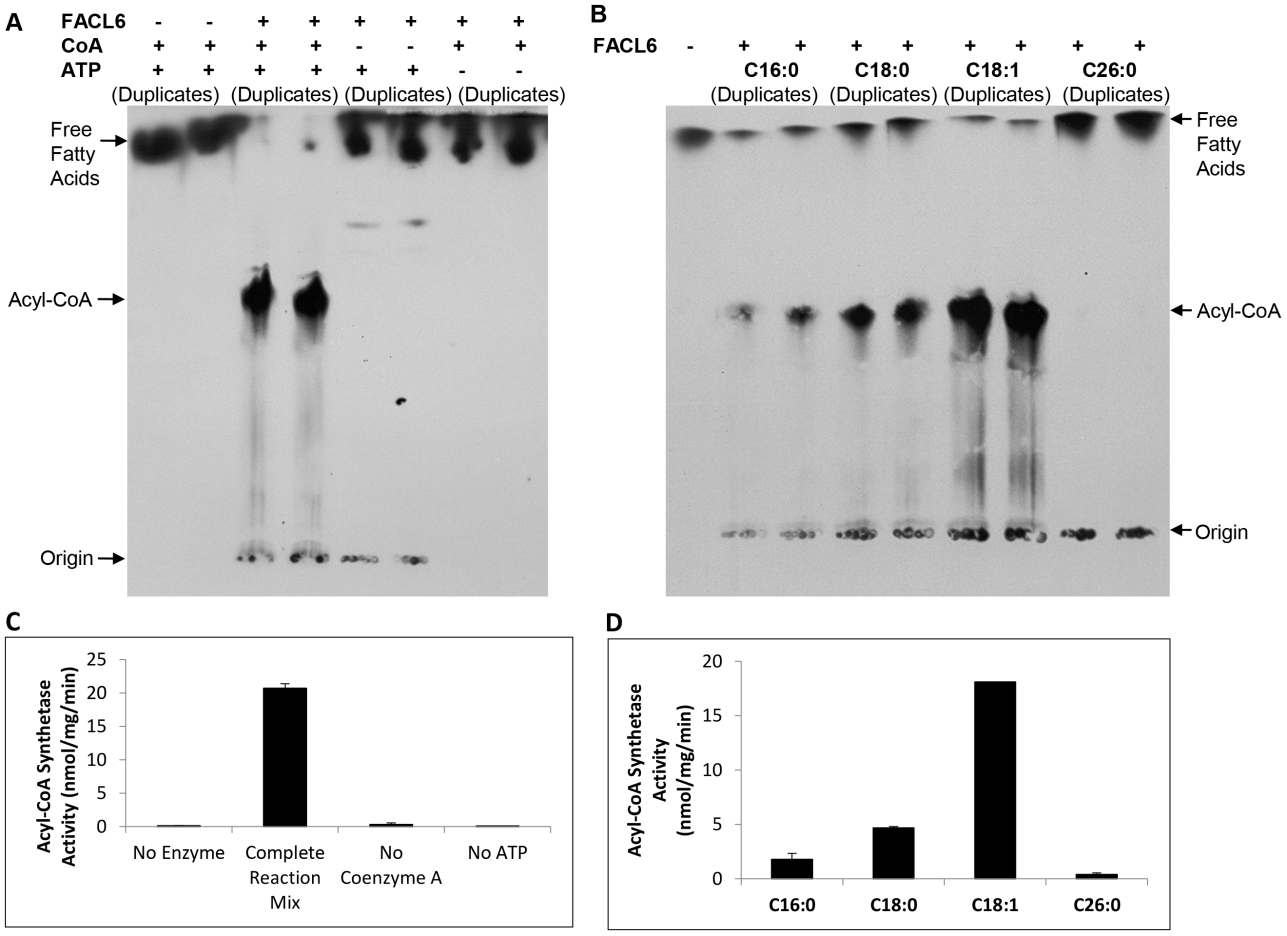
Purified FACL6 displays acyl-coenzyme A synthetase activity with specificity towards oleoyl-CoA. **A,** Purified FACL6 synthesizes acyl-CoA in a CoA- and ATP-dependent manner. Autoradiogram of TLC plate shown. **B,** Acyl-CoA synthetase activity of FACL6 is higher with oleic acid (C18∶1) than with palmitic acid (C16∶0), stearic acid (C18∶0) or hexacosanoic acid (C26∶0). Autoradiogram of TLC plate shown. **C and D, Quantitation of radioactive acyl-CoA bands on TLCs in A and B.** The enzymatic activity of the purified protein was measured using duplicate assays and mean ± SD shown (P<0.005). Radioactive counts were determined by liquid scintillation counting of the silica in the acyl-CoA region of TLC plates in **A** and **B**.

Multiple sequence alignment indicated that FACL6 contained domains homologous to the very long-chain acyl-CoA synthetase domains of eukaryotic FATPs (vertically hatched bars in Fig. 1A). We expressed the FACL6 protein in *E. coli* BL21, purified it from solubilized inclusion bodies and tested whether the purified protein manifested long-chain acyl-CoA synthetase activity, as described under Materials and Methods. As shown in Fig. 1B the 67 kDa histidine-tagged FACL6 was purified to near homogeneity. The purified FACL6 protein displayed acyl-CoA synthetase activity which was dependent on the presence of CoA and ATP (Fig. 2A, C). We tested the acyl chain-length preference of FACL6 protein and, as shown in Fig. 2B, D, we found that the activity of the enzyme was highest with oleic (C18∶1) acid. FACL6 showed lower levels of activity with stearic (C18∶0), palmitic (C16∶0) acids and very low activity with hexacosanoic (C26∶0) acid.

### FACL6 stimulates fatty acid uptake in E. coli cells

**Figure 3:**
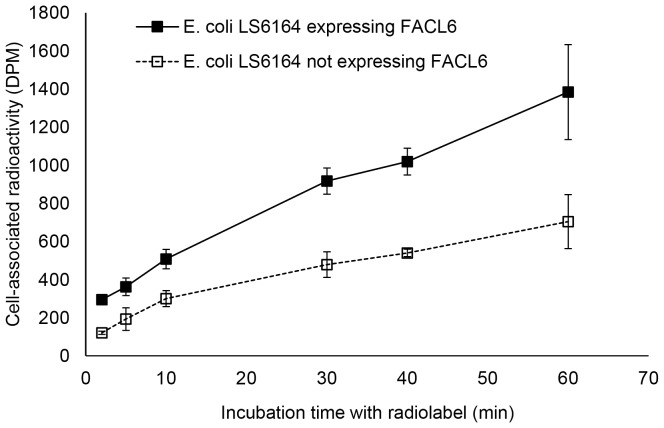
The FACL6 protein stimulates fatty acid uptake in *E. coli*. *E. coli* cells were transformed with a plasmid construct expressing the mycobacterial FACL6 protein. Untransformed *E. coli* was used as negative control. Protein expression was induced with arabinose and the cell numbers of untransformed and transformed cells were equalized by optical density prior to addition of radiolabel. The uptake of radiolabelled oleic acid by intact cells was determined. The radioactivity associated with *E. coli* expressing FACL6 (closed boxes; solid line) was more than 2-fold higher than that associated with cells not expressing FACL6 (open boxes; dashed line). Mean ± SD from three independent experiments is shown (P<0.05).

If FACL6 is involved in the activation of imported fatty acids and trapping them as CoA-esters within the cell, it will act as a metabolic sink for fatty acids entering the cell. So, we examined whether the uptake of fatty acids was stimulated in *E. coli* upon expression of the FACL6 protein. As shown in Fig. 3, the expression of FACL6 increased the levels of radiolabeled oleic acid associated with *E. coli* cells induced with arabinose (for expression of FACL6 protein). Uninduced cells that served as negative controls displayed much lower levels of fatty acid uptake. Radioactive fatty acid associated with *E. coli* cells expressing FACL6 increased with time of incubation with radiolabel and was 2-fold higher than radioactivity associated with control cells not expressing FACL6, at 60 min (Fig. 3). We did not detect significant growth-rate difference in cells expressing the FACL6 protein.

### The FACL6 protein is induced when Mtb enters dormancy

**Figure 4:**
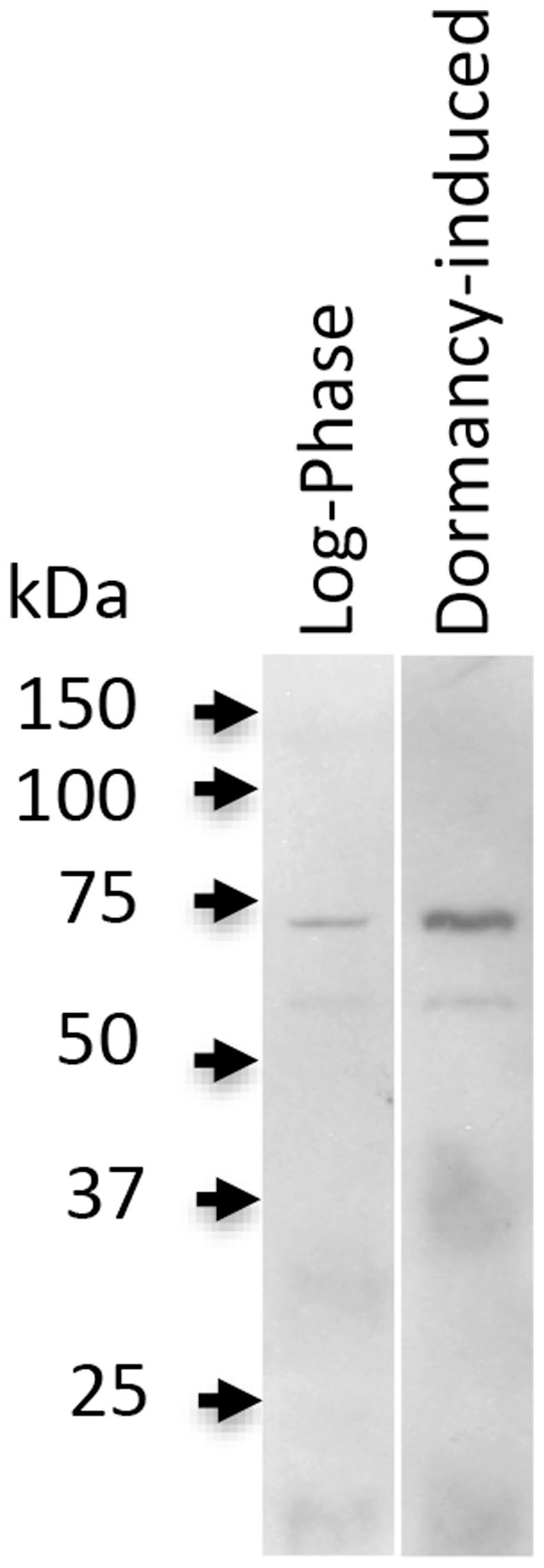
FACL6 protein level is induced in *Mtb* during dormancy-inducing *in vitro* conditions. Lysates of *Mtb* wild type cultures in log-phase or under dormancy-inducing multiple stress condition were analyzed by Western blotting using polyclonal IgG raised against a C-terminal epitope of FACL6 (as indicated in *Materials and Methods*). Loading of log-phase and dormancy-induced samples was equalized using total protein content of each sample as loading control. Two independent experiments were performed and the blot from one experiment is shown.

Fatty acids are the key energy source for *Mtb* during its dormancy and fatty acyl-CoA is a substrate for the TAG-synthesizing enzymes of *Mtb* [3], [4], [19], [26]. Since our results above suggest that FACL6 catalyzes the activation of fatty acid into fatty acyl-CoA, we postulated that it could potentially be involved in TAG synthesis during *Mtb* dormancy. If so, the synthesis of FACL6 protein inside *Mtb* is likely to be induced when it enters dormancy. Therefore, we examined FACL6 protein levels in *Mtb* cell lysates in exponential growth phase and in dormant phase induced by multiple stress [19]. Cell-free extracts from *Mtb* cells in log-phase or subjected to *in vitro* multiple stress conditions, as we have described previously [19], were resolved by SDS-PAGE and analyzed by Western blotting using a polyclonal IgG raised against a C-terminal epitope (EYPEEVSLGRRPQQ) of FACL6 (indicated in Fig. 1). Our results indicate that the expression level of the native 64 kDa FACL6 protein appears to be induced as *Mtb* enters dormancy (Fig. 4). Densitometric analysis of the FACL6 band on the blot showed that the FACL6 protein was induced about 2-fold in dormancy-induced cultures over log-phase cultures.

### Generation of FACL6-deletion mutant of Mtb and its complementation

**Figure 5:**
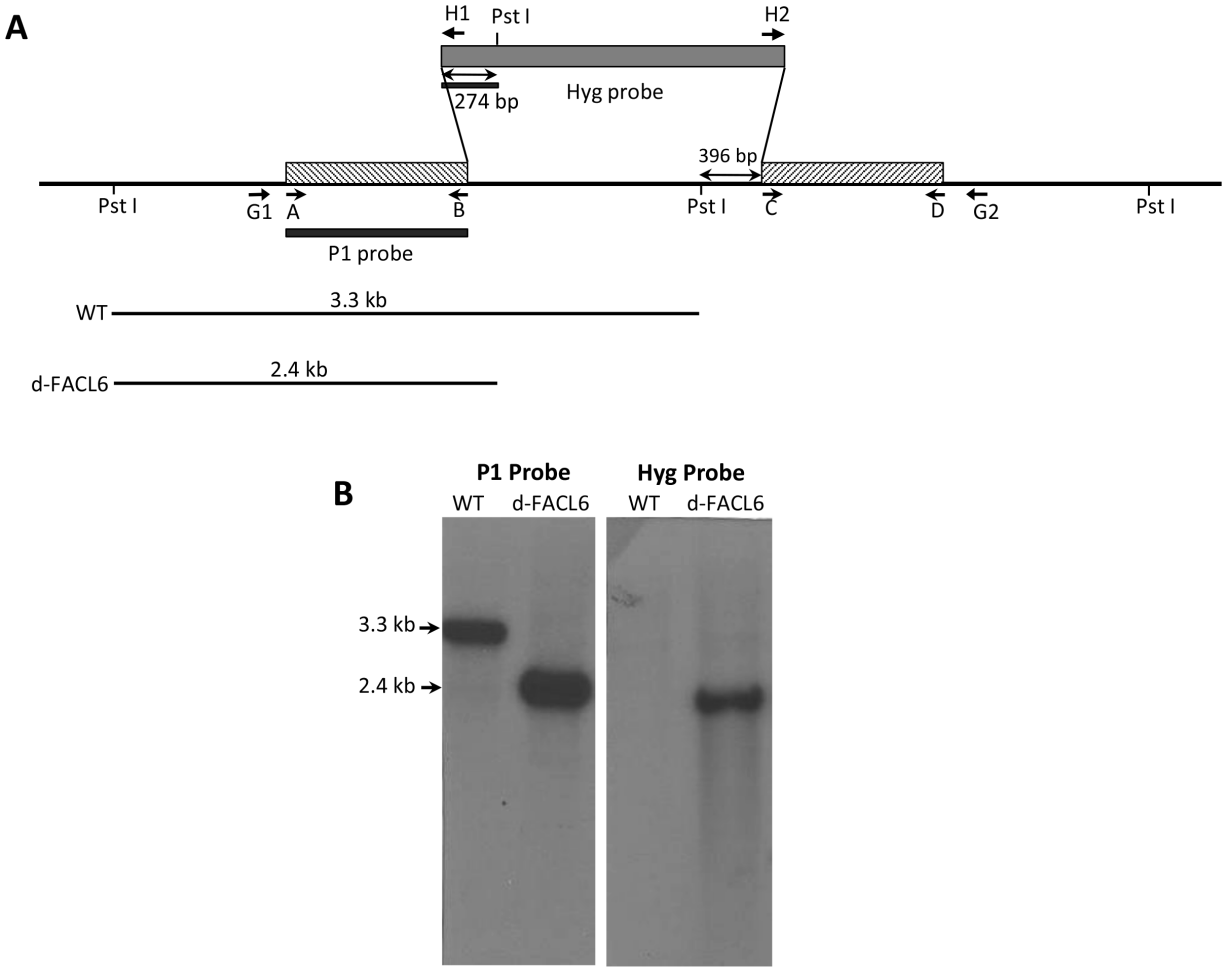
Generation of *FACL6*-deletion mutant of *M. tuberculosis*. (**A**), Schematic depiction shows the genomic locations of the primers and probes used in the construction and confirmation of *facl6* deletion mutants. The sequences of the primers are given in Table 1. (**B**), Genomic DNA from WT *Mtb* and d-*facl6* mutant was digested with PstI and hybridized with the 5′-flank of the *d-facl6* construct as probe and the *hyg* probe. Wild-type genomic DNA digested with PstI and probed with the 5′ flank of the disruption construct yielded a hybridization fragment of 3.3 kb (lane WT). In contrast, PstI digested DNA from the mutant strain showed a smaller band of 2.4 kb due to the presence of a PstI site in the 5′ region of the hyg cassette. Hybridization with the hyg probe showed the expected band in the mutants and no hybridization with the WT DNA.

To assess the role of the *facl6* gene product in TAG production in *Mtb* under stress, we generated a *facl6* gene-knockout mutant by allelic exchange via specialized transduction using the temperature sensitive mycobacteriophage phAE159 as previously described [25]. To prepare the *facl6* disruption construct, a 1572 bp region of the total 1794 bp *facl6* open reading frame was replaced with the hygromycin-resistance gene and was used as the substrate for allelic exchange by double crossover (Fig. 5A). PCR screening of the hygromycin-resistant transductants with a set of primers (d-*facl6*-F and d-*facl6*-R in Table 1), specific for the deleted segment identified several mutants that failed to amplify the expected 720 bp native gene fragment (data not shown). Disruption of *facl6* by double crossover was confirmed by further PCR analysis of the flanking regions (primer pairs G1/H1 and H2/G2), which yielded the expected-size products. Southern blot analysis of *Mtb* wild-type and two *d-facl6* mutants is shown in Fig. 5B. Genomic DNA from *Mtb* digested with PstI showed a 3.3 kb hybridization band when the 5′-flanking region of the construct was used as the probe. DNA from the mutant, under the same conditions, showed a 2.4 kb band due to the presence of a PstI site in the *hyg* gene promoter sequence. Southern blot analysis confirmed that the mutant clone contained a single disrupted copy of the gene. When the same blot was analyzed with the 274 bp probe of the *hyg* promoter gene, the mutant DNA samples yielded a hybridization pattern in agreement with integration by allelic exchange (Fig. 5B). As expected, no hybridization was detected with the wild type DNA sample. We examined whether the *d-facl6* mutant was impaired in dimycoceryl phthiocerol synthesis following procedures we have previously described [27]. The *d-facl6* mutant did not show any alteration in dimycoceryl phthiocerol synthesis from [1-^14^C]propionate labeling experiments indicating that no inadvertent genetic changes had occurred that affect the gene cluster involved in the synthesis of the diester (data not shown). The FACL6 mutant did not display a significant difference in growth when compared to the wild-type in log-phase or in dormant state. The complemented strain had low levels of the FACL6 protein as seen in Western blots of cell lysates (data not shown).

### Loss of FACL6 is associated with a reduced TAG accumulation in Mtb under dormancy-inducing conditions

**Figure 6:**
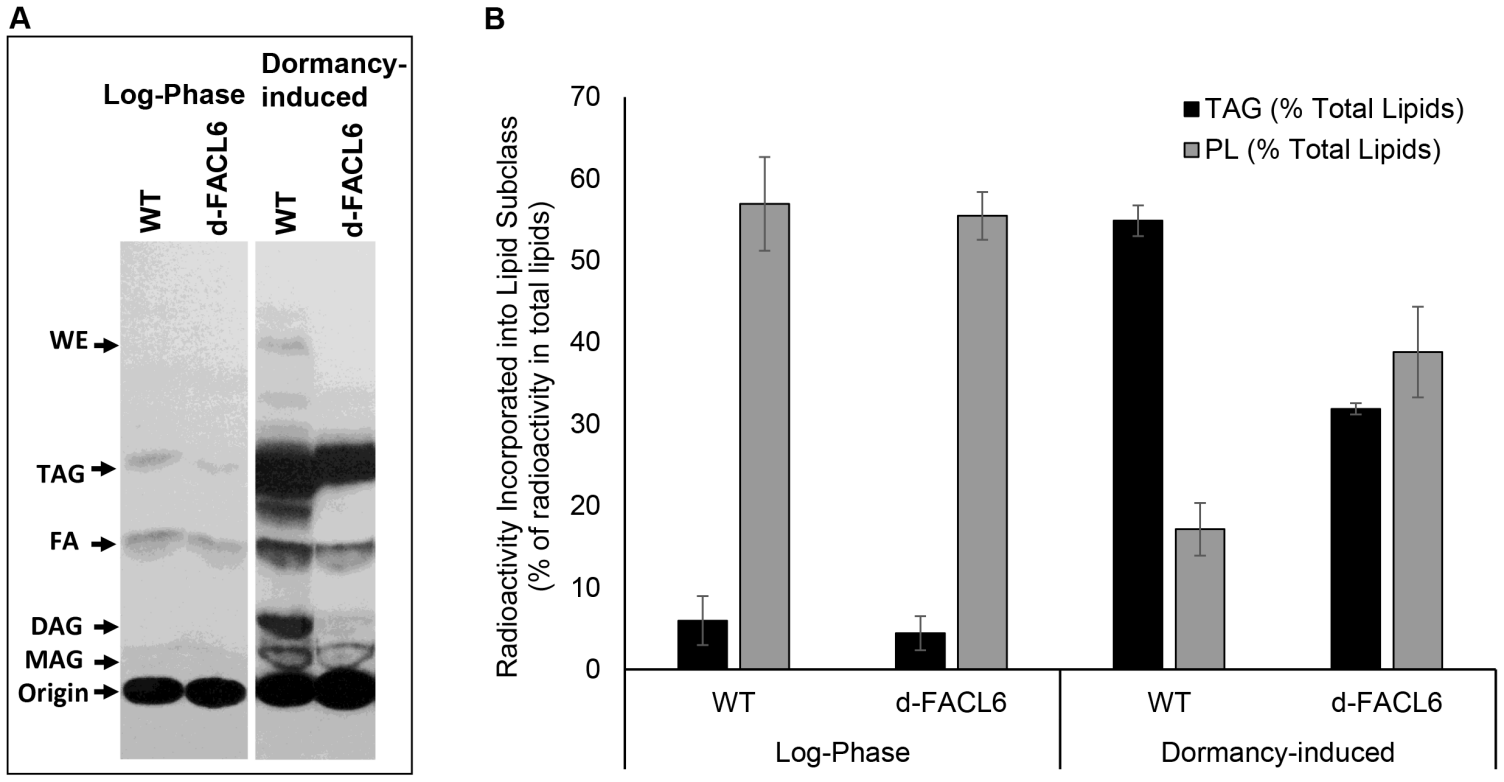
Deletion of FACL6 inhibits triacylglycerol synthesis in *M. tuberculosis* during *in vitro* dormancy. (**A**)**,** ^14^C-Oleic acid incorporation into TAG is inhibited in *Mtb* mutant lacking FACL6. *Mtb* wild type (WT) and *facl6*-deletion mutant (d-FACL6) in exponential (log) phase or subjected to dormancy-inducing multiple-stress conditions were labeled with ^14^C-oleate. Total lipid extracts were resolved on silica-TLC. Autoradiograms of representative TLC plates from one experiment are shown. Relative migrations of authentic lipid standards are indicated. WE, wax esters; TAG, triacylglycerol; FA, fatty acids; DAG, diacylglycerol; MAG, monoacylglycerol; Origin (polar lipids, PL). (**B**), Incorporation of radiolabeled oleic acid into TAG is lower in the *facl6*-deletion mutant compared to WT but incorporation into PL is higher. Radioactivity in bands corresponding to TAG and PL (origin) was determined by scintillation counting and normalized to radioactivity in total lipid extract in respective sample. Three independent experiments performed. Mean ± SD shown (P<0.05).

We have previously shown that *Mtb* enzymes involved in the last step of the TAG synthesis pathway during dormancy utilize fatty acyl-CoA as a substrate along with diacylglycerol [3]. Consequently, an *Mtb* mutant lacking FACL6 with diminished ability to activate fatty acids into CoA-esters might be expected to show decreased TAG synthesis, if FACL6 is involved in dormancy-associated TAG synthesis. Therefore, we subjected *Mtb* wild type and *facl6*-knockout mutant to dormancy-inducing multiple stress conditions in media devoid of Tween-80 but containing Tyloxapol as detergent as we have described earlier [19] and analyzed their ability to incorporate radiolabeled oleic acid into lipids inside the *Mtb* cell. The radioactive counts obtained after incorporation of the radiolabeled oleic acid into total lipids, TAG and polar lipids (PL) for a typical experiment are shown in Table 2. Incorporation of radiolabel into a specific lipid was normalized (as described in Methods) across different samples by expressing the incorporated radioactivity as a fraction of the total radioactivity in the complete lipid extract in the respective sample prior to TLC separation. As shown in Fig. 6A and B, log-phase *Mtb* cells incorporated very low levels of the radiolabel into TAG but dormant *Mtb* cells showed a high level of incorporation of radiolabel into TAG. The deletion of *facl6* resulted in a reduction in the incorporation of exogenously supplied radiolabeled fatty acids into TAG inside dormant *Mtb*. Incorporation of radiolabel into wax esters (WE), diacylglycerol (DAG) and monoacylglycerol (MAG) was also decreased (Fig. 6A). On the other hand, incorporation of radiolabeled oleic acid into polar lipids (origin on TLC plate), which was high in the log-phase wild-type and *facl6*-deletion mutant, was decreased in dormant cells as expected since dormant *Mtb* stops synthesizing polar lipids under multiple-stress conditions that cause it to stop replicating [19]. However, this decrease was much greater in the wild-type and less so in the *facl6*-deletion mutant.

**Table 2:** Metabolic incorporation of ^14^C-oleic acid-derived radioactivity by *Mtb* wild-type and *d-facl6* mutant.

	^14^C-Oleate Incorporation (DPM)		
	Total Lipids	TAG	PL
***Log-Phase WT***	617600±53740	23850±877	374345±3769
***Log-Phase d-FACL6***	627200±56144	18400±1146	359285±3288
***Dormancy-Induced WT***	5225050±466479	2790345±46818	910770±7835
***Dormancy-Induced d-FACL6***	3910550±253214	1222500±31495	1709215±88579

*Mtb* wild-type and *d-facl6* mutant in log-phase or subjected to dormancy-inducing conditions were incubated with radiolabel as described in Materials and Methods. Radioactivity (DPM, disintegrations per minute) in total lipid extracts was determined by counting dried aliquots by scintillation counting. Radioactivity in triacylglycerol (TAG) and polar lipids (PL) was determined by liquid scintillation counting after TLC separation of total lipid extracts.

**Figure 7:**
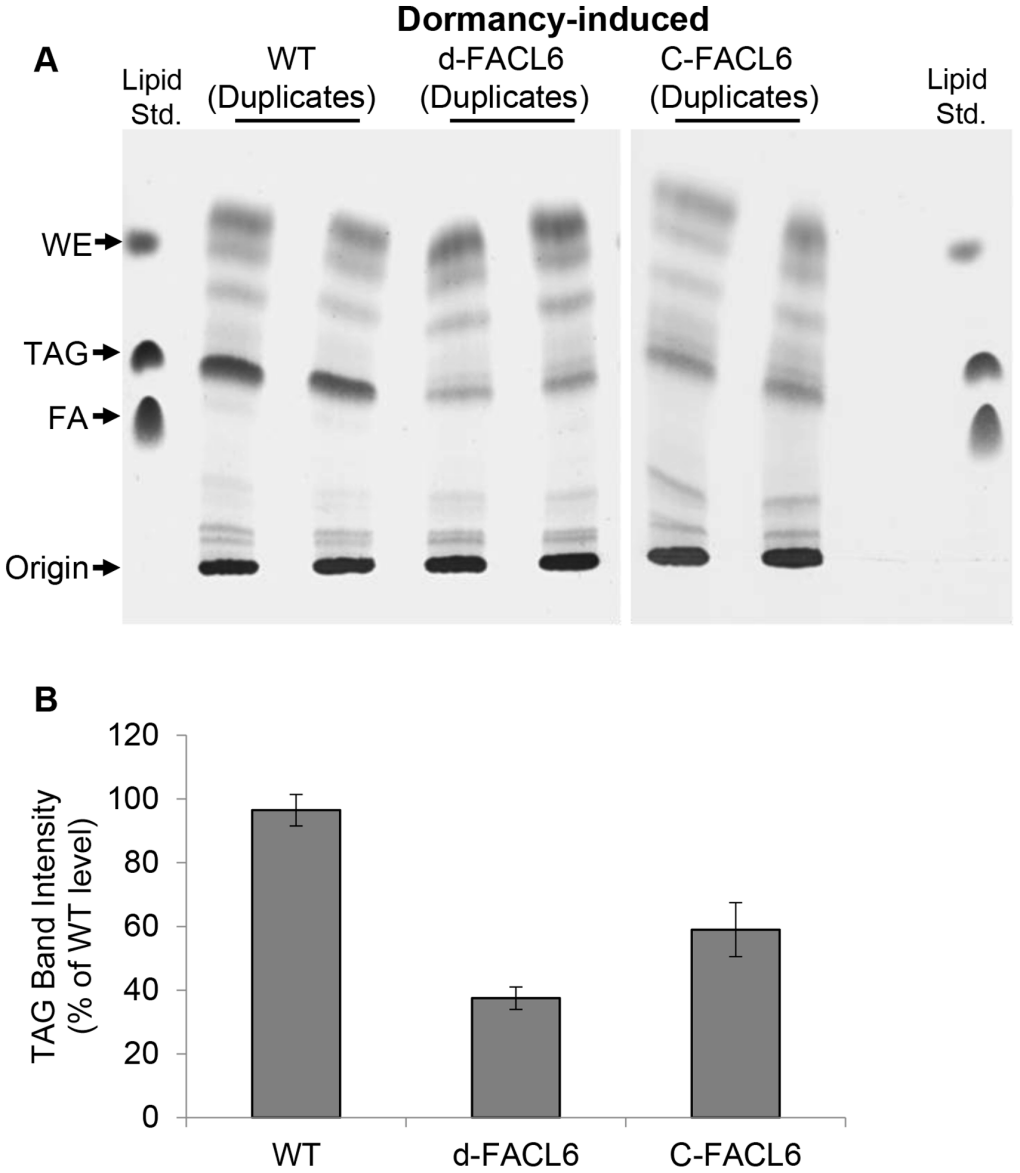
*Mtb* mutant lacking FACL6 is inhibited in dormancy-associated TAG accumulation and complementation partially restores lost phenotype. (**A**), *Mtb* wild type (WT), FACL6-deletion mutant (d-FACL6) and complemented mutant (C-FACL6) were fed with 100 µM oleic acid under dormancy-inducing multiple-stress condition. Lipids were extracted, resolved on silica-TLC and visualized by charring as described in Materials and Methods. A typical TLC plate with samples loaded in duplicate and lipid standards at the left and right edges of TLC plate is shown. (**B**), Quantitation of the charred TAG band intensity by densitometry in silica-TLC shows that the d-FACL6 mutant accumulates only about 40% of WT TAG levels. TAG band intensity was normalized using optical density of cultures. Three independent experiments were performed. Mean ± SD shown (P<0.05).

We investigated whether the loss in the ability to incorporate radiolabeled fatty acids into TAG in the *facl6*-deletion mutant was also reflected in its ability to accumulate TAG reserves using exogenously supplied fatty acids under dormancy-inducing conditions. *Mtb* cells were subjected to multiple stress and incubated with 100 µM non-radiolabeled oleic acid to allow for intracellular TAG accumulation. We found that the dormancy-associated accumulation of TAG inside *Mtb* was also inhibited in the *facl6*-deletion mutant and this decrease in TAG accumulation was partially restored in the complemented mutant (Fig. 7A and B).

### Acyl-CoA synthetase activity is diminished in cell-free extracts of FACL6 mutant of Mtb

**Figure 8:**
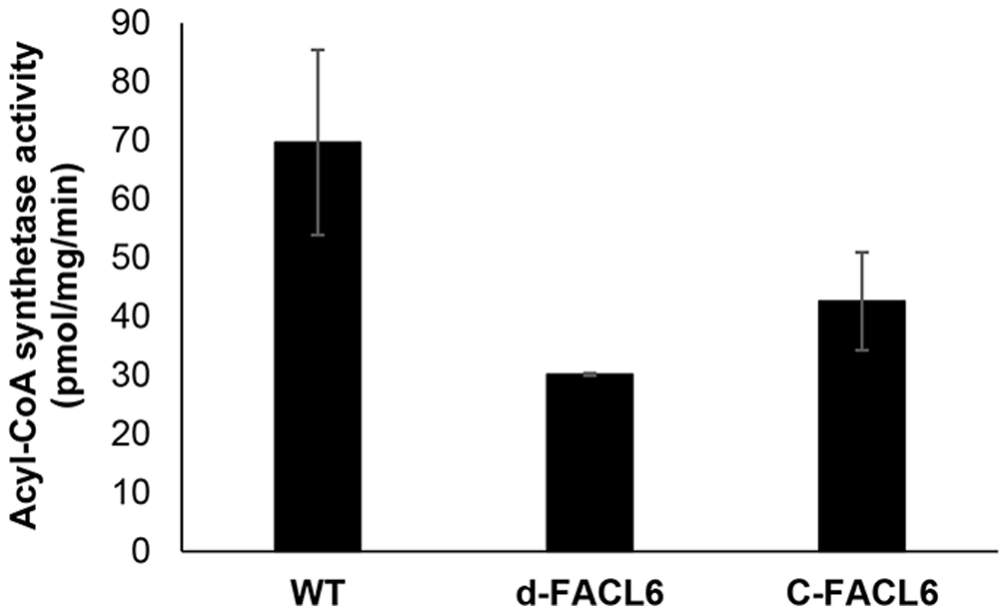
Oleoyl-coenzyme A synthetase activity is decreased in the dormancy-induced *M. tuberculosis* mFATP1-deletion mutant. Cell-free lysates prepared from *Mtb* wild type (WT), FACL6-deletion mutant (d-FACL6) and complemented FACL6 mutant (C-FACL6), subjected to dormancy-inducing conditions, were assayed for acyl-CoA synthetase activity as described in Methods. Radioactivity partitioned in aqueous phase after incubation was taken as a measure of enzyme activity. Experiment was repeated independently three times and mean ± SD from duplicates in a representative experiment shown.

Since the purified FACL6 protein showed acyl-CoA synthetase activity, we investigated whether deletion of the *facl6* gene in *Mtb* affected acyl-CoA synthetase activity levels in the *Mtb* cell. We assayed cell-free extracts of *Mtb* wild-type, FACL6 deletion mutant and complemented FACL6 mutant subjected to dormancy-inducing multiple-stress for acyl-CoA synthetase activity using radiolabeled oleic acid, ATP, CoA and Mg^2+^ as described under Materials and Methods. We found that the acyl-CoA synthetase activity level in the FACL6-deletion mutant was significantly lower than that in the wild-type (Fig. 8). Complementation partially restored the lost phenotype.

## Discussion

Fatty acids, which play essential and diverse roles in various life processes, need to be activated by formation of acyl-CoA in order for them to be utilized in anabolic and catabolic pathways. To participate in such pathways, fatty acids need to traverse membranes of cells and sub-cellular vesicles. There are two schools of thought regarding the mechanisms involved in the transport of fatty acids. According to the biophysical model, the “flip-flop” of fatty acids across membranes by adsorption and passive diffusion is sufficient to meet even the most demanding needs for fatty acids in cellular processes. The rate-limiting step is the desorption of fatty acids from the membrane. But according to the other model, specific fatty acid transport proteins are a necessary part of this process [13].

Several *Mtb* gene products involved in the activation of fatty acids have been studied. *Mtb* has 34 FACL-like genes and a subset of them have been found to function as fatty acyl-AMP ligases that activate long-chain fatty acids as acyl-adenylates which are transferred to polyketide synthases for further extension of the acyl chain [18]. The crystal structures of the acyl-AMP ligase FadD28 and acyl-CoA ligase FadD13 revealed that an insertion motif involved in the formation of acyl adenylates in some of the fatty acyl-AMP ligases of *Mtb* was absent in the acyl-CoA ligase [28], [29]. However, FadD10, which is an acyl-AMP ligase does not contain the insertion motif [30]. FadD13, a peripherally membrane-associated acyl-CoA ligase, was shown to activate C26 and C24 fatty acids for use by the *mymA* operon of *Mtb* in the synthesis of cell envelope under acidic conditions [31], [32]. The acyl-CoA ligase FadD5, which is located within the *mce1* operon of *Mtb* was shown to be involved in growth of *Mtb* on mycolic acids as the sole carbon source [33]. FadD3 was demonstrated to function as an acyl-CoA ligase involved in cholesterol catabolism [34]. FadD26 was shown to be involved in phthiocerol and phthiodiolone dimycocerosate biosynthesis and FadD22 and FadD29 were involved in phenolic glycolipid biosynthesis [35]. The activities of several FadD proteins of *Mtb* was shown to be regulated by the cAMP-dependent protein lysine acetyltransferase of *Mtb* but FACL6 was not one of them [22].

Our current study has focused on the only *Mtb* FACL protein identified to be homologous to mammalian FATP1 [12]. The mammalian FATP1 was the first protein to be implicated in the uptake and transport of fatty acids across membranes [36]. Since FATP1 and other homologous proteins have not been consistently localized to the plasma membrane, an alternative mechanism has been proposed in which fatty acids are metabolically trapped inside the cell by converting them to CoA-esters [13]. Subsequently, other researchers identified another protein, Very Long-Chain Acyl-CoA Synthetase (ACSL), which was most homologous to FATP1 but was involved in the activation of very long-chain fatty acids (containing ≥22 carbons) [37].

The FACL6 protein contains regions with strong amino acid identities with the AMP-binding domain and very long-chain acyl-CoA synthetase domain of mammalian FATP (Fig. 1). Therefore we examined the ability of the purified FACL6 protein to activate fatty acids by esterifying them to coenzyme A in the presence of ATP. Our observations of the acyl-CoA synthetase activity of FACL6 (Fig. 2) supports its potential role in the uptake of fatty acids by trapping them metabolically as CoA esters. Our findings showing that FACL6 is able to increase the levels of association of radiolabeled fatty acid with *E. coli* cells (Fig. 3) further supports this hypothesis. Like the mammalian counterpart, FACL6 is predicted to be a cytoplasmic protein by *in silico* analysis (PSLPred; http://www.imtech.res.in/raghava/pslpred; accessed July 2014). In order to perform its function, FACL6 does not need to be embedded in the membrane. A peripheral association with the membrane may be sufficient. Further studies are needed to determine whether FACL6 might be peripherally membrane-associated like the *Mtb* fatty acyl-CoA synthetase, FadD13, whose structure and sub-cellular localization were reported recently [32]. Our results also suggest that FACL6 is induced as *Mtb* enters a dormant state (Fig. 4). We found that the acyl-CoA synthetase activity level was significantly lower in the FACL6-deletion mutant than that in the wild-type in log-phase (data not shown) and in multiple-stressed *Mtb* and complementation partially restored the lost activity (Fig. 8).

Complementation of inducible genes presents complications with respect to the choice of promoters to be used. In the present case, to test whether complementation of *facl6*, which might be involved in dormancy, can restore the phenotype of the deletion mutant we used Hsp60 promoter-driven expression of *facl6*. The decrease in TAG was only partially restored by complementation. An attempt to complement the deletion mutant with a vector that should drive *facl6* gene expression with its own promoter (0.5 kb and 1 kb 5′-flank sequence) also failed to fully restore TAG accumulation (data not shown).

In mammalian systems, different isoforms of ACSLs and FATPs are involved in channeling fatty acids into catabolic or anabolic pathways and certain members have been shown to preferentially channel fatty acids into the biosynthesis of different lipid classes [15], [16]. The human FATP1 was shown to channel exogenously supplied fatty acids into TAG synthesis and downregulate sphingomyelin and cholesterol metabolism [17]. More recently in another study, the expansion of lipid droplets in *Caenorhabditis elegans* was directly linked to the synergistic association between FATP1 and DGAT2 proteins at the endoplasmic reticulum-lipid droplet interface [38]. In another report, the role of human FATP2a/ACVSL1 in the preferential channeling of exogenous n-3 fatty acids into acyl-CoA pools destined for phosphatidylinositol biosynthesis was demonstrated [39]. The same authors have very recently shown that FATP2 plays an important role in linking fatty acid transport and intracellular fatty acid trafficking in a manner which is dependent on the level of FATP2 expression [40]. The mammalian ACSL5 was shown to mediate fatty acid channeling between anabolic and catabolic pathways in hepatic cells [41]. Such findings in mammalian FATP/ACSL proteins hint at the possibility that FACL6, which displays acyl-CoA synthetase activity in our assays, might also play important roles in metabolic channeling of fatty acids inside the *Mtb* cell.

We have previously shown that the *Mtb* TGS enzymes that catalyze the final acylation of DAG to TAG utilize long-chain fatty acyl-CoA as substrate [3]. In this study, we observed that the loss of FACL6 caused a decrease in TAG, diacylglycerol, monoacylglycerol and wax ester synthesis inside *Mtb* under dormancy-inducing conditions (Fig. 6). The biosynthesis of all these neutral lipid subclasses requires acyl-CoA which is a product of FACL6. Since the deletion of FACL6 decreases TAG synthesis/accumulation and increases polar lipid synthesis/accumulation in *Mtb* (Figs. 6, 7), we propose that FACL6 possibly plays an important role in the channeling of fatty acids into TAG for use by *Mtb* during its dormancy. It is likely that other *fadD* genes of *Mtb* might also be involved in the activation of fatty acids for dormancy-associated TAG synthesis. Further studies are needed to determine the identity of such *fadD* gene products.

The results we have obtained suggest that FACL6 is probably involved in the activation of fatty acids that are imported by *Mtb* from host lipid sources. FACL6 may also play an important role in channeling the imported fatty acids into specific intracellular pool(s) within *Mtb* for use by TAG synthetic pathways, which play a key role in *Mtb* dormancy. Further studies are needed to ascertain such a possible role for FACL6, which would make it an important target for developing drugs against dormant *Mtb*.
